# Priming with a “simplified regimen” of HIV-1 DNA vaccine is as good as a “standard regimen” when boosted with heterologous HIV-1 MVA vaccine

**DOI:** 10.1186/1742-4690-9-S2-P108

**Published:** 2012-09-13

**Authors:** P Munseri, A Kroidl, C Nilsson, C Moshiro, S Aboud, A Joachim, C Geldmacher, E Aris, D Buma, E Lyamuya, F Gotch, K Godoy-Ramirez, K Pallangyo, L Maboko, M Marovich, M Robb, M Hoelscher, M Janabi, P Mann, S Joseph, S Mfinanga, W Stoehr, F Mhalu, B Wahren, G Biberfeld, S McCormack, E Sandstrom, M Bakari

**Affiliations:** 1 Muhimbili University of Health and Allied Sciences, Dar es Salaam, Tanzania, United Republic of; 2 Department for Infectious Diseases and Tropical Medicine, Munich, Germany; 3 The Swedish Institute for Communicable Disease Control, SMI, Stockholm, Sweden; 4 Division for Infectious Diseases and Tropical Medicine, Germany; 5 Imperial College, UK; 6 Swedish Institute of Communicable Disease Control (SMI), Stockholm, Sweden; 7 Mbeya Medical Research Programme, Tanzania, United Republic of; 8 Walter Reed Army Institute of Research (WRAIR), USA; 9 The Henry M. Jackson foundation, USA; 10 MRC, Clinical Trials Unit, UK; 11 National Institute for Medical Research, Tanzania, United Republic of; 12 Karolinska Institutet, Sweden; 13 Swedish Institute for Communicable Disease Control (SMI), Sweden; 14 MRC Clinical Trials Unit, UK; 15 Venhalsan, Karolinska Institutet, Sodersjukhuset, Sweden

## Background

Intradermal priming with DNA prior to MVA boosts gives strong and broad immunogenicity, however that required 5 injections at each immunization. A higher concentration of DNA might allow a simpler administration.

## Methods

This double blind, placebo-controlled trial, enrolled 120 (12 placebo) HIV-uninfected volunteers, in Dar es Salaam and Mbeya. Two pools of DNA plasmids were used (pool1 EnvABC + RevB, pool2 GagAB + RTB) boosted with MVA CMDR EnvE, GagA, PolA. Volunteers were randomized in three groups of 40, primed with either two injections of 300ug, one in each arm, (total 600ug) of DNA with combined plasmids (group IA) or two injections of 300ug with one pool in each arm (total 600ug) of DNA (group IIA) “simplified regimens” or five injections, 2 (pool1) and 3 (pool2) injections in the right and left arm respectively, (total 1000ug) of DNA (IIIA) “standard regimen”. DNA/Placebo priming was administered by a needlefree (Biojector) device intradermally at weeks 0, 4 and 12. All volunteers were boosted intramuscularly with 10 pfu of recombinant MVA/placebo at weeks 30 and 46. The primary end point was the number of ELISpot responders to Gag and Env, 2 weeks post the last vaccination.

## Results

There were no safety concerns. The response rate to Gag and/or Env was 27/32 (84%) in group IA vs 31/33 (94%) in group IIA (p=0.26). The response rate to Gag and/or Env when comparing the ‘simplified regimens’ vs ‘standard’ regimen was 58/65 (89%) vs 32/32 (100%) p=0.09. In responders the median magnitude (IQR) response to Gag was 165 (100,365) SFC/million PBMC vs 210 (120,320), p=0.46 while the magnitude for Env was 150 (92,225) vs 110 (80,160) p=0.17 for the ‘simplified’ vs ‘standard’ regimens.

## Conclusion

The simplified HIV-1 DNA regimens primed as well as the standard regimen for cellular immune responses following boosting with with MVA.

